# Development of expression-based biomarkers of Dasatinib response in hematologic malignancies

**DOI:** 10.1038/s41408-017-0013-z

**Published:** 2017-12-15

**Authors:** Monica K. Akre, Amit Mitra, Wen Wang, Chad L. Myers, Brian Van Ness

**Affiliations:** 1 Department of Molecular, Cellular, Developmental Biology, and Genetics, Masonic Cancer Center, University of Minnesota, Minneapolis, MN 55455 USA; 2 Department of Computer Science and Engineering, University of Minnesota, Minneapolis, MN 55455 USA

Numerous collections of cancer cell lines provide opportunities to characterize genetic signatures that distinguish response and resistance to a variety of drugs. The NCI-60, Cancer Cell Line Encylopedia (CCLE), and the Genomics of Drug Sensitivity in Cancer (GDSC) provide a wealth of information that includes genomic sequences, mutational status, gene expression, as well as response to panels of drugs used in cancer therapies^1–3^. This offers a unique opportunity to apply approaches that may provide genetic profiles that define response and resistance, with the potential to apply these as predictors in clinical decisions.

The GDSC is a collection of 1047 cell lines from diverse tumor types that have been tested with 265 drugs^1^. The data collection includes DNA sequence, mutation status, and gene expression data that we have used to develop a pipeline of computational approaches that predict response and resistance. Drug response is determined by a 9 step twofold serial dilution of drug concentration and measuring cell viability. From these, two quantitative values are provided: the drug concentration required to reduce viability by 50% (IC50) and the area under the dose-response survival curve (AUSC). Gene expression data is available from the Affymetrix U219 gene array platform.

Because expression patterns may vary widely simply based on tissue specificity, we chose to limit our initial analysis to B-cell malignancies, represented by 71 cell lines derived from leukemias, lymphomas, and myelomas. Our approach comprises a series of steps in which we classify response and resistance, develop a differential classification profile of gene expression patterns, identify features by pathway analysis, and validate on cell lines and reported clinical outcomes. We demonstrate this approach to stratify and predict response to the protein tyrosine kinase inhibitor, dasatinib.

Dasatinib is a multi-target kinase inhibitor that has affinity for about 50 kinases and is most widely used to manage chronic myelogenous leukemia^4, 5^. For this report, we develop an approach that identifies a five gene signature distinguishing dasatinib response and resistance.

We arranged the 71 B-cell lines by response, using both Area Under the Survival Curve (AUSC) and IC50. The distribution of response favored non-response, with only 14 lines showing a strong response to low doses, and 11 lines showing essentially no response. Specifically, we classified Responders as lines that show an AUSC < 0.75, and an IC50 value less than the maximum drug concentration divided by 4 and Non-Responders were defined as having an AUSC > 0.98 and an IC50 greater than maximum dose tested. This resulted in the 14 strong Responder lines vs. 11 highly resistant (Non-Responder) lines, representing the extreme ends of response and resistance. Our rationale was that underlying this wide separation of response may be a common expression signature or pathway(s) that can serve as a predictive biomarker.

Differential gene expression between the Responder and Non-Responder lines was performed using Significance Analysis of Microarray (see Supplementary Methods), with a false discovery rate limited to 10%. This resulted in 228 genes to further analyze for their relevance to the dasatinib response.

The B-cell-receptor (BCR) pathway has been demonstrated to be active at different stages of B-cell development^6–8^. Activation of various oncogenes and tumor suppressor genes gives rise to the malignancies at different stages of B-cell development (Fig. 1). The distribution of response to dasatinib along the B-cell differentiation path indicates cancers arising from a pre- or early- B-cell may be more likely to respond than B cells or plasma cells later in development that notably have decrease expression of BCR pathway genes.

**Fig. 1:**
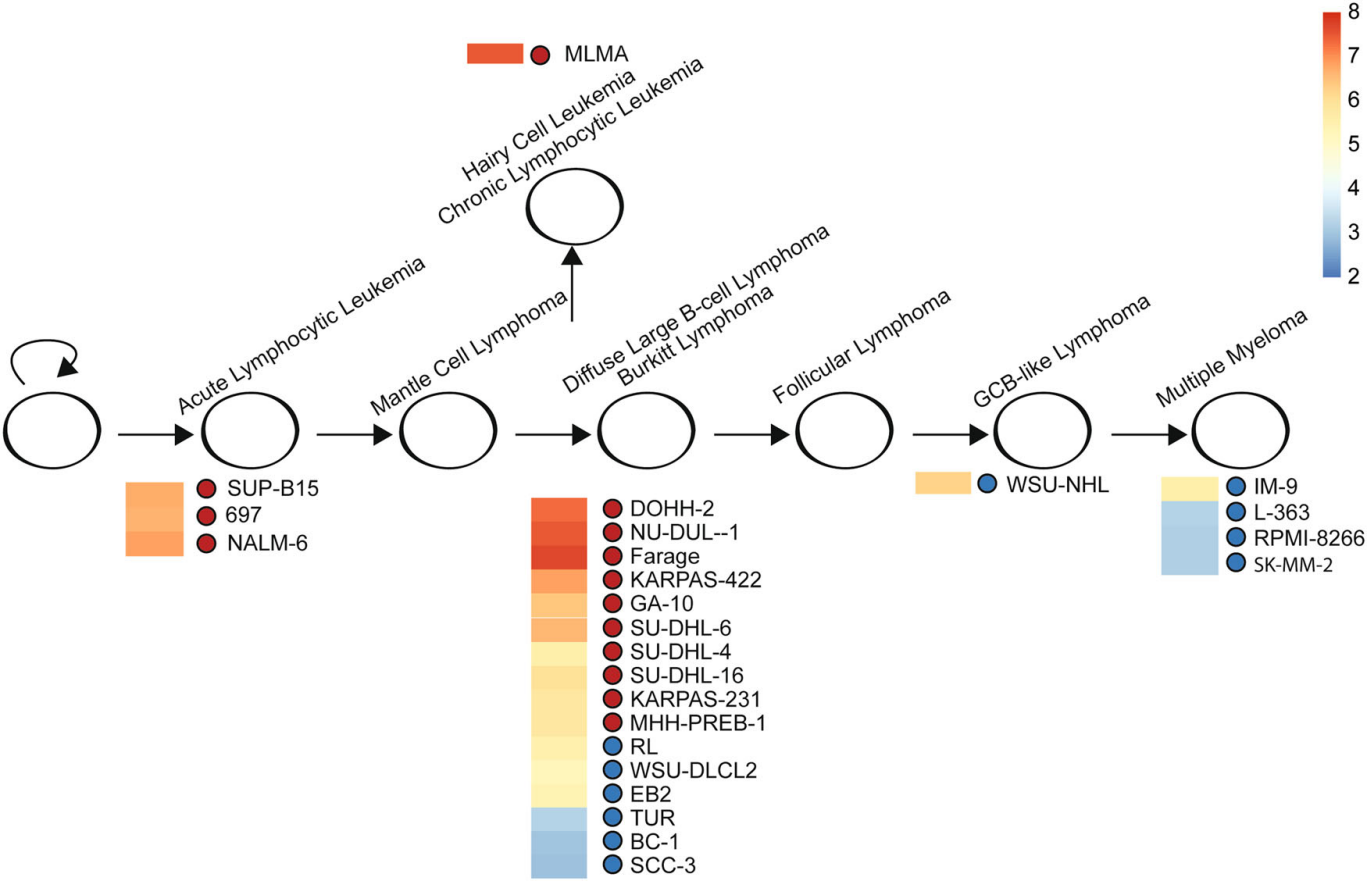
B-cell differentiation stages with malignancy and CD19 expression of cell lines Maturation of B-cells is represented from left to right. Malignancies arising from corresponding stages of B-cell development are depicted along with cell lines representing those malignancies in the same vertical axis. Next to cell line names are either a red dot (Responders) or blue dot (Non-Responders) along with their corresponding log2 fluorescence intensity of CD19, the marker found most associated with dasatinib response

Pathway analysis was conducted using ingenuity pathway analysis (Qiagen) on the 228 differentially expressed genes and their log-fold ratio data of Responders relative to Non-Responders. Notably, the top canonical pathway with a significant *z*-score was the BCR pathway (*p*-value = 0.013). Using the log-fold ratio scores of Responders relative to Non-Responders, molecules of the BCR pathway reflected a uniquely activated pattern in the Responders (Fig. 2a).

**Fig. 2:**
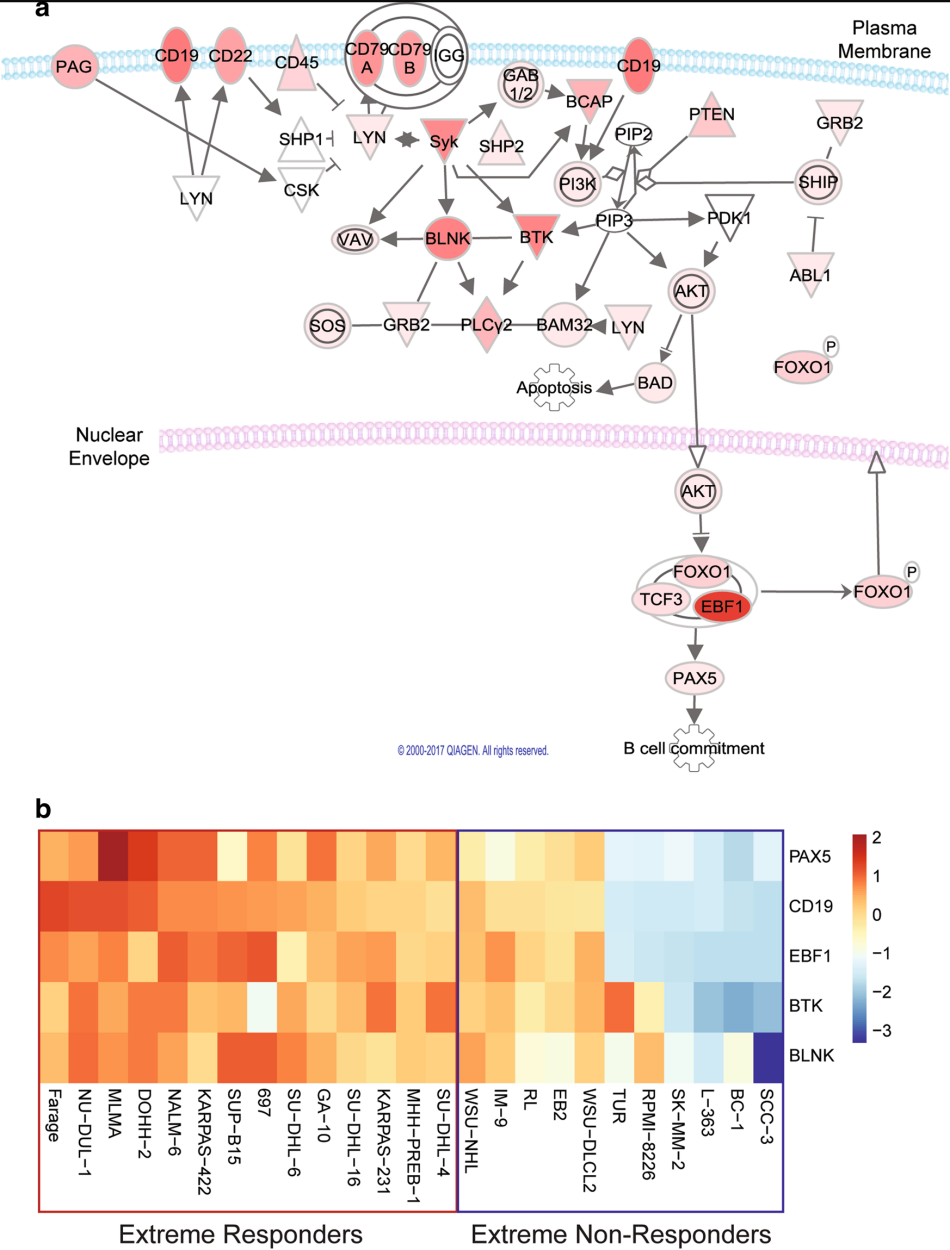
BCR pathway activated in extreme responders **a** Genes of the BCR pathway represented as expression ratios of highly sensitive Responders relative to Non-Responders. The darker the red-colored molecule represents a greater fold-change between groups. **b** Gene expression signature that discriminates Responders from Non-Responders Cell lines are listed along the *x*-axis while the five genes most associated with dasatinib response are on the *y*-axis. Expression values are represented as scaled as *z*-scores of the log2 transformed fluorescence intensities. The 14 extreme Responders are boxed in red on the left of the heatmap, the 11 extreme Non-Responders are boxed in blue on the right. The dynamic ranges of each gene in the signature is not always reflective of its contribution to identifying response as can be seen in the case of PAX5. This gene is highly significant (*p* < 0.0001) in differentiating response, but its absolute values vary subtly

We further characterized the differentially expressed genes of the BCR pathway between the Responders (high expression) and Non-Responders (low expression). The genes from the BCR pathway were analyzed using a Mann–Whitney t-test between Responders and Non-Responders. Genes that were significant (*p*-value < 0.05) were further analyzed. This resulted in the identification of five genes (Fig. 2b).

Intermediate response groups were included to identify an expression trend across the full range of responses. We reasoned that effective drug-response predictors would also show a linear trend between Responders, intermediate groups, and Non-Responders. These groupings included cell lines with a partial response (AUSC between 0.75–0.85) and limited response (AUSC between 0.85–0.98). The five genes displaying the best separations were chosen for use in a predictive scoring system described below. Analysis of variances were performed using all four groupings and indicated 4 of the 5 genes showed highly significant differences between the Responders and Non-Responders, and trends of decreasing expression across the increasing resistance groupings.

Eleven cell lines not included in the differential expression analysis had available gene expression data (eight from CCLE, three from GDSC), but not dasatinib response data. These were tested in-house for dasatinib response. The CCLE/GDSC expression platforms were then normalized to one another to obtain comparable values (not shown). During the validation phase, we used the AUSC as the sole metric indicating response defining a Responder (AUSC < 0.8) and Non-Responder (AUSC > 0.8).

A simple algorithm was developed based on average expression values of the Responders and Non-Responders for each of the five genes, which we refer to as Response Averages (RA). Lines that had expression lower than the RA of CD19 were immediately binned as a Non-Responder. Lines that had expression higher than that of the CD19 RA were given a score of “1” for each remaining gene (EBF1, PAX5, BTK, or BLNK) for which the expression exceeded the RA. If a cell line’s score was less than 3, they were binned as Non-Responders. This scoring system accurately predicted the response of 9 of the 11 previously untested lines.

As described above, the discriminating genes were originally determined using the extreme Responders vs. the extreme Non-Responders. When applying the scoring system to the intermediate responding lines (that were not included in the gene discrimination modeling), 30 lines were correctly predicted for an accuracy rate of 67%. It should be noted low CD19 does place 23 out of 23 of these cell lines in the Non-Responder category. Thus, low CD19 is an effective discriminator of non-response. Nevertheless, the five gene discrimination was very accurate in distinguishing the highly sensitive from the highly resistant lines. Despite the difficulty of determining the response of the equivocal intermediate group, the sensitivity of the test set, training set, and intermediate group is 78.9% and specificity is 74.6%.

MM lines (plasma cells) rarely express CD19, and show a low activation of the BCR pathway. Thus, our BCR gene discrimination model would indicate poor response. Indeed, a recent clinical study (NCT00429949) of relapsed, refractory, or plateau phase MM patients was discontinued after using dasatinib as a single agent in which a partial response occurred in only 1 of 21 enrolled in the study.

Waldenström’s macroglobulinemia (WM) is also a plasma cell malignancy, but in contrast to multiple myeloma, is CD19+, and has recently been described to express an activated BCR pathway^9^. WM primary patient lines exhibited good response to dasatinib in primary patient samples (*n* = 32)^9^ supporting our findings that the expression of CD19 and four other molecules of the BCR pathway are associated with dasatinib response.

Mantle cell lymphoma (MCL) is mid-stage B-cell malignancy and may, or may not, express CD19^10, 11^. Kim et al.^12^ showed acquired bortezomib resistance (BTZ-R) was accompanied by a re-activation of the BCR pathway. Along with this re-activation, a collateral sensitivity to dasatinib was observed.

We wondered whether this relationship between bortezomib and dasatinib would also be seen in the U266 multiple myeloma line. U266-P and U266-VR (Velcade resistant) was developed in our laboratory and had RNAseq data available^13^. The U266-VR line had a 2.8-fold increase in FPKM reads for CD19. In addition, the U266-P line had a dasatinib AUSC value of 0.91, whereas U266-VR had an AUSC of 0.73. These data further support that CD19 and the BCR pathway are consistent biomarkers associated with B-cell lineage cells response to dasatinib.

We show here that cell line expression and drug response can be interrogated through differential expression and pathway analysis to find meaningful relationships and identify biomarkers of drug response.

This is just one example of the use of available data bases to develop response signatures. Similar approaches may provide gene signatures across many other drugs within the GDSC, CCLE, or similar large cell line data bases.

## Electronic supplementary material

Supplementary Methods

## References

[1] Yang W (2012). Genomics of drug sensitivity in cancer (GDSC): A resource for therapeutic biomarker discovery in cancer cells. Nucleic Acids Res.

[2] Barretina J (2012). The cancer cell line encyclopedia enables predictive modelling of anticancer drug sensitivity. Nature.

[3] Shoemaker RH (2006). The NCI60 human tumour cell line anticancer drug screen. Nat. Rev. Cancer.

[4] Shi H, Zhang CJ, Chen GYJ, Yao SQ (2012). Cell-based proteome profiling of potential Dasatinib targets by use of affinity-based probes. J. Am. Chem. Soc..

[5] Keating GM (2017). Dasatinib: A review in chronic myeloid leukaemia and Ph+ Acute Lymphoblastic Leukaemia. Drugs.

[6] Irish JM, Czerwinski DK, Nolan GP, Levy R (2006). Altered B-cell receptor signaling kinetics distinguish human follicular lymphoma B cells from tumor-infiltrating nonmalignant B cells. Blood.

[7] Irish JM (2010). B-cell signaling networks reveal a negative prognostic human lymphoma cell subset that emerges during tumor progression. Proc. Natl Acad. Sci..

[8] Blix ES (2012). Phospho-specific flow cytometry identifies aberrant signaling in indolent B-cell lymphoma. BMC Cancer.

[9] Argyropoulos K (2016). Clonal B cells in Waldenström’s macroglobulinemia exhibit functional features of chronic active B-cell receptor signaling. Leukemia.

[10] Molot RJ (1994). Antigen expression and polymerase chain reaction amplification of mantle cell lymphomas. Blood.

[11] Ginaldi L (1998). Levels of expression of CD19 and CD20 in chronic B cell leukaemias. J. Clin. Pathol..

[12] Kim, A., Seong, K. M., Kang, H. J., Park, S., Lee, S. -S. Inhibition of Lyn is a promising treatment for mantle cell lymphoma with bortezomib resistance. *Oncotarget* [Internet]. **6**. Available from: www.impactjournals.com/oncotarget 10.18632/oncotarget.5425 PMC4741995 26517678

[13] Mitra A (2017). A gene expression signature distinguishes innate response and resistance to proteasome inhibitors in multiple myeloma. Blood Cancer J.

