# Supplementary material for: Development of expression-based biomarkers of Dasatinib response in hematologic malignancies
Source: Blood Cancer J. 2017 Dec 15;7(12):652. doi: 10.1038/s41408-017-0013-z (PMC5802564; doi:10.1038/s41408-017-0013-z)
Supplement: Supplementary file 1 — Supplementary Methods [file 41408_2017_13_MOESM1_ESM.docx]

**Supplementary Methods**

**Expression Analysis**

Robust-Multi-Array Average (RMA) normalized expression data and drug response data was downloaded directly from the GDSC website (www.cancerrx.org).

CCLE Robust-Multi-Array Average (RMA) normalized expression data was downloaded from Genomicscape.com. Multiple ENSEMBL IDs mapping to the same gene region were averaged.

**Normalization of CCLE values GDSC**

There are 48 cell lines of B-cell origin that are in both the GDSC and the CCLE. These were used to determine the best method of normalization. Lowess plot comparison between these 48 lines showed the best concordance between the two sets. First, the natural log of each CCLE intensitiy value was subtracted from that value, then the set was quantile normalized to the GDSC. The Bioconductor limma package normalizeQuantile function was modified to adjust the CCLE values to their GDSC “twin” lines(17).

**Code Availability**

R scripts available by request.

**Statistical Analysis**

The Significant Analysis of Microarray (sam) function of the siggenes R package was used in differential expression analysis (18,19). The thresholds were varied until the number of genes exceeded 200, yet still within an FDR of 0.10.

Responders and Non-Responders expression values for each gene in the signature were compared using the unpaired, nonparametric, Mann-Whitney test in GraphPad (Prism).

Ordinary one-way ANOVA was performed in GraphPad (Prism) on all genes of signature using 4 groupings based on response values: Responders (AUSC<0.75), Partial Responders (AUSC 075-0.85) and Limited Responders (AUSC 0.85-0.98), and Non-Responders (AUSC>0.98).

The cor() function of the R stats package was used to find Pearson correlation coefficients using all expression values between cell lines (20).

All heatmaps, including the Pearson correlation coefficients were rendered using the pheatmap package (21).

**Tissue Culture**

All cell lines were maintained in RPMI-1640 (Lonza) supplemented with 10% FBS (Invitrogen Life Technology), 1X Antibiotic/Antimycotic (Gibco), 1X L-Glutamine (Gibco), and 1ng/mL of human IL-6. Incubators were humidified and maintained at 37° Centigrade with a 5% CO_2_ content.

Cells were plated at a concentration of 5x10^5^/mL on day0. On day 1, a dilution series of concentrations (2-fold dilutions from the max dose of 5.12μM) as well as a DMSO vehicle control were administered in triplicate. On day 4 (72 hours after treatment) cell viability was measured by Cell Titer-Glo Luminescent cell viability assay according to manufacturer's instructions (Promega) and luminescence was read and recorded using Synergy 2 Microplate Reader (Biotek).

Maximum viability assigned, as 100% for IC50 or 1 for AUSC calculations, was normalized to untreated controls. IC_50_values were estimated by calculating the nonlinear regression using the inhibitor-normalized response equation (variable slope) in GraphPad (Prism).

As done in the calculation of AUSC in the GDSC, wells containing media, drug, but no cells were used to calculate the value for normalizing maximum response, which corresponds to a 0 value. AUSC used the concentrations that overlapped with GDSC doses and substituted the first lowest concentration within the GDSC dilution series for the lower 2 doses tested in lab. AUSC were calculated using GraphPad (Prism).

­­
